# An integrated dataset on organisational culture, job satisfaction and performance in the hospitality industry

**DOI:** 10.1016/j.dib.2018.04.137

**Published:** 2018-05-04

**Authors:** Joy Dirisu, Rowland Worlu, Adewale Osibanjo, Odunayo Salau, Taiye Borishade, Sandra Meninwa, Tolulope Atolagbe

**Affiliations:** Department of Business Management, Covenant University, Nigeria

## Abstract

The relevance of organisational culture on job satisfaction and performance particularly within the hospitality sector cannot be over-emphasized. The culture of an organization goes a long way in distinguishing it from other organizations because it shows its ability to either be successful or to fail. To however achieve excellence and high-level performance, it is important to note that for effective and efficient operation, an organization would need a formal approach of communication as well as for making decisions and completing the tasks to match the needs of the organization. The managerial implications drawn from the study is that organizations should take advantage of their culture and inculcate values that will enhance performance.

## Specification Table

**Table:** 

**Subject area**	Business Management
**More Specific Subject Area:**	Organizational Behaviour and HRM
**Type of Data**	Primary data (Table and Figure)
**How Data was Acquired**	Researcher-made questionnaire analysis
**Data format**	Raw, analyzed, Inferential statistical data
**Experimental Factors**	Sample comprises selected hotels in Nigeria. The researcher-made questionnaire which contained data on organisational culture, job satisfaction and performance
**Experimental features**	The importance of organisational culture transcends its role in improving the quality of decisions. It affects employees’ attitudes, values, behaviour, competencies, communication process, productivity and competitiveness in the long term.
**Data Source Location**	Lagos, Nigeria
**Data Accessibility**	Data is included in this article

## Value of data

• The data can be used by managers to properly make decisions that in the long-run would lead to goal attainment in the organization.
• The data can be used to enlighten managers on the importance of organisational culture and how it can be beneficial to overall performance of the organization.
• The data provides ample knowledge on how different organisational culture can interact effectively by building diverse dimensions of interaction that brings about the creation of a conducive and encouraging organisational climate and culture that affects the way members of an organisation work or function.
• The data described in this article is made widely accessible to facilitate critical or extended analysis.

## 1 Introduction

Organisational culture refers to certain characteristics that shape how human beings behave and communicate within any organisational setting. The concept of organisational culture is of fundamental interest among individuals, groups, and organisations as they try to understand how the culture of an organisation can make a difference or have a sway on the satisfaction and performance of all members of the organisation especially in the hospitality industry.

## 2 Data

The data comprised raw inferential statistical data on the effect of organisational culture and job satisfaction on the performance of selected hotels in Nigeria. The study population of this research comprises managers and supervisors of selected six (6) hotels from a list of 131 hotels adjudged to be the top performing/most popular hotel brands in Lagos State by Tripadvisor (2017). The information presented spread across the six hotels used in this research work. 205 copies of questionnaire were retrieved from managers and supervisors of Southern Sun, Wheatbaker, West view, Radisson Blu Anchorage, Westland and Royal view hotels. The items in the questionnaire were adopted from Organisational Cultural Profile (OCP) developed by [1], [3], [4] who illustrated five categories: employee orientation (EO), innovation (IN), customer focus (CF), systematic and management control (SMC) and social responsibility (SR). While job satisfaction and performance were measured based on modified indicators from prior studies [2].

**Fig. 1:**
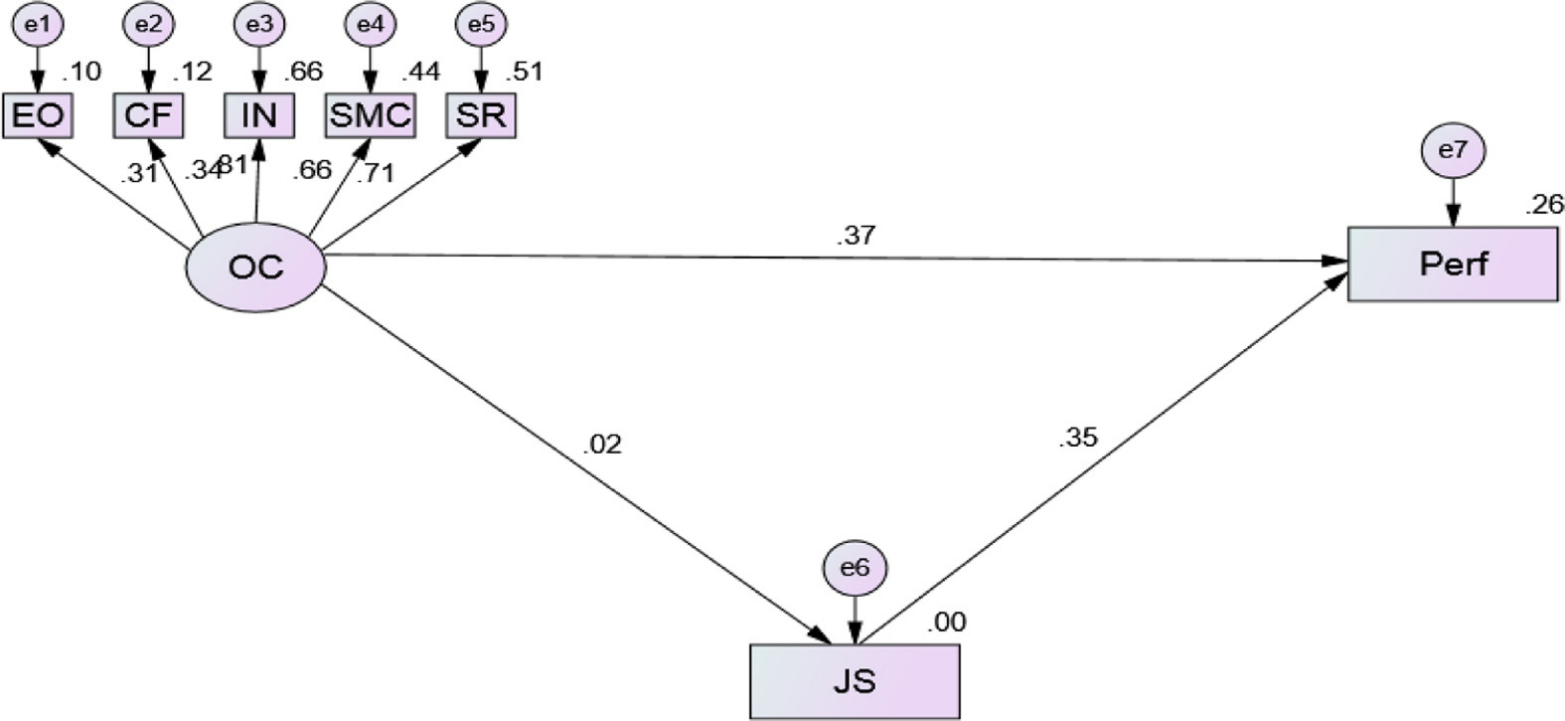
Standardized regression weights.

Source: Field Survey, 2017.

Explicitly, a proposed framework model has been tested using structural equation modelling (SEM) to show the relationship between observed and unobserved variables. A model fit was evaluated by examining several fit indices which include: chi-square (*χ*2), chi-square/degree of freedom (*χ*2/d*f*), Goodness-of-Fit Index (GFI), Comparative Fit Index (CFI), Standardized Root Mean Residual (SRMR) and Root Mean Square Error of Approximation (RMSEA). Having run the test, the SEM was obtained, and results of fit indices as presented in Fig. 1 and Table 1, Table 2 respectively.

**Table 1:** The model fit summary showing the goodness of fitness.

Goodness of fit	SEMs value	Recommendation values	Remarks
Chi­Square/Degree of Freedom (CMIN/DF)	2.462	≤ 3.00	Acceptable fit
Normed Fit Index (NFI)	.967	≥ .90	Good fit
Comparative Fit Index ( CFI)	.921	≥ .90	Very Good fit
Incremental Fit Index (IFI)	.984	≥ .90	Good fit
Root Mean Squared Error of Approximation (RMSEA)	.039	≤ .08	Good fit
Goodness of Fit (GFI)	.977	≥ .90	Good fit

**Table 2:** Regression weights.

DV		IV	Estimate	S.E.	C.R.	P	Remark
JS	<---	OC	.017	.230	2.293	.010	Sig
EO	<---	OC	.308	.435	3.636	***	Sig
CF	<---	OC	.340	.346	4.080	***	Sig
IN	<---	OC	.813	.548	5.243	***	Sig
SMC	<---	OC	.660	.539	5.111	***	Sig
SR	<---	OC	.712	.655	5.179	***	Sig
Perf	<---	OC	.366	.320	4.355	***	Sig
Perf	<---	JS	.352	.045	7.582	***	Sig

## 3 Experimental design, materials and methods

The data presented was based on a quantitative study. A descriptive research design was adopted in this study to obtain the opinions of managers and supervisors on how organisational culture has influenced job satisfaction and the extent to which it influences overall performance of selected hotels.

Survey method was considered appropriate as data collection method based on the fact that it allows for the collection of standardized data that permits the researcher to produce information for answering the how, who, what and when questions regarding the subject matter. Managers and Supervisors of The Wheatbaker Hotel, Ikoyi; Southern Sun Hotel, Ikoyi; Radisson Blu Anchorage Hotel, Victoria Island; Royal View Hotel and Suites, Mafoluku-Oshodi; Westland Hotels and Suites, Ikotun and West View Hotel, Mafoluku-Oshodi were selected for the study.

The use of primary source of data (questionnaire) was used for collecting data from a cross section of customers across sample hotels. The study employed a combination of structured and unstructured question items. The collected data were coded and entered into SPSS version 22. Data analysis was done; using Statistical Package for Social Sciences-22. Although Statistical Package for Social Sciences may be limited when it comes to advanced modeling and development of statistical approaches.

Statistical Package for Social Sciences makes in-depth data analysis quicker because the programme knows the location of the cases and variables. It also comes with more procedures of screening the information in preparation for further analysis. More importantly, Statistical Package for Social Sciences is designed to make certain that the output is kept separate from the data itself particularly because it stores all results in a separate file that is different from the data. Data was analyzed using inferential statistical tests which involved the use of structural equation modelling (SEM) to tests hypotheses about relationships between variables.

## 4 Ethical considerations

The researchers ensured that respondents were well informed about the background and the purpose of this research and they were kept abreast with the participation process. Respondents were offered the opportunity to stay anonymous and their responses were treated confidentially.

## 5 Academic and managerial implications

This study revealed that organizational culture has significant and positive impact on *job satisfaction and performance particularly within the hospitality sector*. The requisite for hospitality industry to develop internal structures as complex as the environments in which they operate becomes necessary on a continual basis. Hence, this culture and structure must continually be directed towards the need to satisfy and balance internal needs and to adapt to environmental circumstances. This present study has extensive implications for both the hospitality sector, managers, researchers and undergraduate students in this regard. To this end, the data presented in this article is imperative for more comprehensive analysis or investigation.
