# Supplementary material for: An integrated dataset on organisational culture, job satisfaction and performance in the hospitality industry
Source: Data Brief. 2018 May 4;19:317–21. doi: 10.1016/j.dib.2018.04.137 (PMC5992975; doi:10.1016/j.dib.2018.04.137)
Supplement: Supplementary file 1 — Supplementary material [file mmc1.docx]

**DECLARATION OF INTEREST FORM**

AN INTEGRATED DATASET ON ORGANISATIONAL CULTURE, JOB SATISFACTION AND PERFORMANCE IN THE HOSPITALITY INDUSTRY

**Joy Dirisu;** Covenant University

joy.dirisu@covenantuniversity.edu.ng

**Rowland Worlu;** Covenant University

rowland.worlu@covenantuniversity.edu.ng

**Adewale Osibanjo;** Covenant University

adewale.osibanjo@covenantuniversity.edu.ng

**Odunayo Salau;** Covenant University

odunayo.salau@covenantuniversity.edu.ng

**Taiye Borisade;** Covenant University

taiye.borisade@covenantuniversity.edu.ng

**Sandra.Meninwa;** Covenant University

[chukwufumnayameninwa@yahoo.com](mailto:chukwufumnayameninwa@yahoo.com)

**Tolu Atolagbe;** Covenant University

tolu.atolagbe@covenantuniversity.edu.ng

We, the Authors of paper entitled above certify that we have seen and approved the final version of the manuscript being submitted. This is an original work and has not received prior publication and is not under consideration for publication elsewhere. It is also important to state that there is no financial/personal interest or belief that could affect our objectivity and to prevent ambiguity, we humbly want to state explicitly that theire is no conflicts of interest as regrads the review and publication of this paper.

Thank you.

DIRISU Joy

*Signed*
